# Ligand and antagonist driven regulation of the *Vibrio cholerae* quorum-sensing receptor CqsS

**DOI:** 10.1111/j.1365-2958.2012.07992.x

**Published:** 2012-02-14

**Authors:** Yunzhou Wei, Wai-Leung Ng, Jianping Cong, Bonnie L Bassler

**Affiliations:** 1 Department of Molecular Biology, Princeton UniversityPrinceton, NJ 08544, USA; 2 Howard Hughes Medical InstituteChevy Chase, MD 20815, USA

## Abstract

Quorum sensing, a bacterial cell–cell communication process, controls biofilm formation and virulence factor production in *Vibrio cholerae*, a human pathogen that causes the disease cholera. The major *V. cholerae* autoinducer is (*S*)-3-hydroxytridecan-4-one (CAI-1). A membrane bound two-component sensor histidine kinase called CqsS detects CAI-1, and the CqsS → LuxU → LuxO phosphorelay cascade transduces the information encoded in CAI-1 into the cell. Because the CAI-1 ligand is known and because the signalling circuit is simple, consisting of only three proteins, this system is ideal for analysing ligand regulation of a sensor histidine kinase. Here we reconstitute the CqsS → LuxU → LuxO phosphorylation cascade *in vitro*. We find that CAI-1 inhibits the initial auto-phosphorylation of CqsS whereas subsequent phosphotransfer steps and CqsS phosphatase activity are not CAI-1-controlled. CAI-1 binding to CqsS causes a conformational change that renders His194 in CqsS inaccessible to the CqsS catalytic domain. CqsS mutants with altered ligand detection specificities are faithfully controlled by their corresponding modified ligands *in vitro*. Likewise, pairing of agonists and antagonists allows *in vitro* assessment of their opposing activities. Our data are consistent with a two-state model for ligand control of histidine kinases.

## Introduction

Two-component systems (TCS) are ubiquitous in bacteria and are used to adapt to environmental changes (Krell *et al*., 2010). A typical TCS system consists of a sensor histidine kinase and a response regulator (Casino *et al*., 2010). Sensor histidine kinases usually contain N-terminal transmembrane sensing domains, dimerization histidine phosphotransfer (DHp) domains and C-terminal catalytic ATP-binding (CA) domains (Dutta *et al*., 1999). In response to environmental signals, the CA domain catalyses the phosphorylation of a specific histidine residue in the DHp domain using ATP as the phospho-donor. This phosphoryl group is subsequently transferred to a particular aspartate residue in the receiver domain of the cognate response regulator, regulating its DNA binding activity, enzymatic activity or protein–protein interactions (Lupas and Stock, 1989; Weiss *et al*., 1992; Takai *et al*., 2006; Yoshida *et al*., 2006; Petrova and Sauer, 2009; Paul *et al*., 2011). Histidine kinases can also possess a phosphatase activity that removes the phosphoryl group from the response regulator (Igo *et al*., 1989; Yoshida *et al*., 2002; Huynh *et al*., 2010). Some of these sensory systems, called phosphorelay systems, include additional modules that exist as separate proteins or are contained within the sensor histidine kinases (Hoch, 2000). In phosphorelay systems, the phosphorylated residues are denoted H1 → D1 → H2 → D2. Following phosphorylation on the sensor histidine kinase (H1) and subsequent phosphotransfer to a response regulator module (D1), in a phosphorelay system, the phosphoryl group is sequentially transferred to a histidine on a histidine-containing phosphotransfer (HPt) module (H2), and finally to an aspartate on another response regulator module (D2). Presumably, the additional sites of phosphorylation increase the potential points at which regulation can occur or enable signal integration from multiple sensory pathways (Hoch, 2000).

**Fig. 1:**
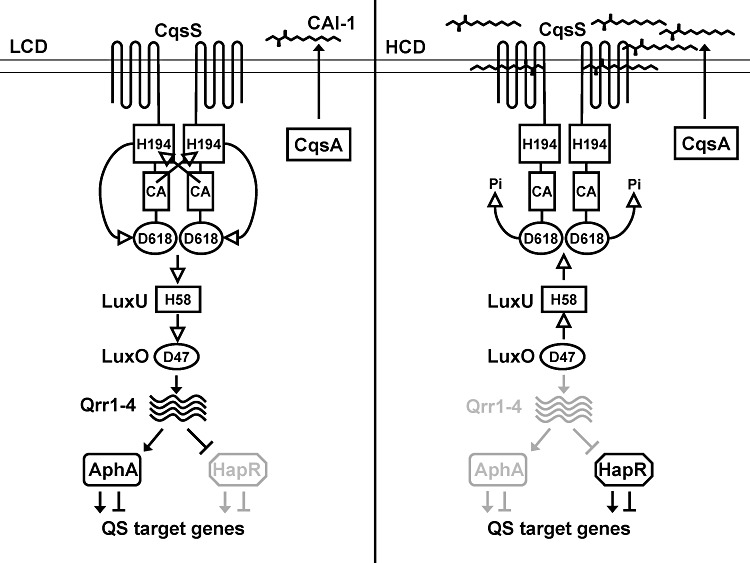
The *V. cholerae* CqsS/CAI-1 quorum-sensing phosphorelay system. CqsA synthesizes CAI-1, which is (*S*)-3-hydroxytridecan-4-one and CqsS is the CAI-1 receptor. At low cell density (LCD, left), when CAI-1 concentration is below the detection limit, CqsS functions as a kinase. Following auto-phosphorylation at His194, the phosphoryl group is transferred to Asp618 on the CqsS receiver domain. The next transfer is to His58 on LuxU. LuxU, in turn, transfers the phosphoryl group to Asp47 on LuxO. Once phosphorylated, LuxO activates the transcription of genes encoding four small regulatory RNAs called Qrr1-4. Qrr1-4 activates the translation of AphA and represses the translation of HapR. AphA regulates genes that are beneficial for individual behaviours. At high cell density (HCD, right), CAI-1 accumulates, binds CqsS, and switches CqsS to a phosphatase. Phospho-flow is reversed and LuxO is dephosphorylated. Qrr1-4 are not produced, and HapR protein is translated. HapR regulates genes that promote group behaviours. Open arrows denote phospho-flow and closed arrows denote gene regulation and CAI-1 synthesis. We note that the *trans* phosphorylation of CqsS His194 is shown for simplicity based on data from other TCS systems. We do not exclude the possibility of *cis* phosphorylation. In this study, the reverse phosphate flow from LuxU to CqsS that occurs at high cell density is termed the CqsS phosphatase activity for continuity with previous reports. We note that the canonical definition of phosphatase activity in a two-component system refers to that of a histidine kinase targeting the aspartyl-phosphate on the partner response regulator.

We are interested in phosphorelay systems involved in quorum sensing (QS). QS is a bacterial cell–cell communication process that relies on the production, release and detection of extracellular signalling molecules called autoinducers (Ng and Bassler, 2009). This process enables populations of bacteria to synchronize gene expression in response to fluctuations in population density. QS ensures that collective behaviours such as bioluminescence, biofilm formation and virulence factor production are only performed at cell densities that enable them to be effective (Bassler *et al*., 1994; Miller *et al*., 2002; Greenberg, 2003). In *Vibrio cholerae*, two QS autoinducer/phosphorelay pathways, CAI-1/CqsS and AI-2/LuxPQ converge to regulate gene expression (Miller *et al*., 2002). The CAI-1/CqsS system, the focus of this study, is the major QS system in *V. cholerae*. The autoinducer CAI-1 is (*S*)-3-hydroxytridecan-4-one (Higgins *et al*., 2007; Wei *et al*., 2011). Detection of and response to CAI-1 relies on a phosphorelay system in which CqsS is the receptor for CAI-1 (Miller *et al*., 2002). The membrane bound sensor histidine kinase CqsS contains the typical transmembrane/sensing domain, DHp domain, and CA domain (H1). In addition, CqsS contains a C-terminal receiver domain (D1). At low cell density, when the concentration of CAI-1 is below the threshold for detection, CqsS functions as a kinase and auto-phosphorylation on His194 (H1) occurs. Following phosphotransfer to Asp618 (D1) on the CqsS receiver domain, the phosphoryl group is transferred to His58 on LuxU (H2), an HPt protein. Phosphorylated LuxU, in turn, transfers the phosphoryl group to Asp47 on the response regulator LuxO (D2) (Freeman and Bassler, 1999a, b). Phosphorylated LuxO activates the transcription of genes encoding four small regulatory RNAs Qrr1-4 (Lenz *et al*., 2004), which promote the translation of *aphA* and inhibit the translation of *hapR* (Lenz *et al*., 2004; Rutherford *et al*., 2011). AphA and HapR are the master quorum-sensing regulators in *V. cholerae* at low and high cell densities respectively (Fig. 1, left). Thus, at low cell density, AphA protein is made and HapR is not. AphA controls the regulon of genes involved in individual cell behaviours. At high cell density, when CAI-1 has accumulated, CqsS binds to CAI-1. This event switches CqsS to a phosphatase and reverses the flow of phosphate through the circuit resulting in dephosphorylation of LuxU and LuxO. Transcription of *qrr*1-4 ceases, which frees the *hapR* mRNA for translation and terminates the activation of translation of *aphA* mRNA. Thus, at high cell density, HapR protein is produced and AphA protein is not. HapR regulates downstream target genes that underpin group behaviours (Fig. 1, right). Traits controlled by QS in *V. cholerae* include biofilm formation and virulence factor production (Zhu *et al*., 2002; Hammer and Bassler, 2003).

Elegant studies of bacterial TCSs have revealed their importance and many of their functions (Casino *et al*., 2009; Martin *et al*., 2009). However, biochemical studies of membrane bound sensor histidine kinases like CqsS have been plagued by the following issues. First, the identities of the ligands for most TCSs are not known. Thus, it has not been possible to study ligand regulation of these sensory circuits *in vitro*. Second, structural studies of membrane bound proteins are notoriously difficult and therefore structural analyses of TCS have been limited to sensors with well-defined periplasmic domains (Neiditch *et al*., 2005; 2006; Cheung and Hendrickson, 2010; Tajima *et al*., 2011). The CqsS pathway provides an excellent opportunity for detailed biochemical studies because the ligand CAI-1 is known, CAI-1 is membrane permeable making it possible to study the receptor–ligand interactions in membrane vesicles, and because the circuit is simple; only three proteins are involved, CqsS, LuxU and LuxO. In this report, we reconstitute the complete CqsS QS phosphorylation cascade with full-length membrane bound CqsS receptor and purified LuxU and LuxO and we show that the circuit is controlled by CAI-1. Phosphorylation of His194 on CqsS (H1), rather than phosphotransfer or phosphatase activity is the step that CAI-1 controls. CAI-1 functions by rendering His194 inaccessible to the CA domain. We also characterize antagonist action using this *in vitro* system. The signal transduction events we quantified using this reconstituted system can be explained by a two-state theoretical framework for histidine kinases.

## Results

### Reconstitution of the CqsS phosphorylation cascade *in vitro*

**Fig. 2:**
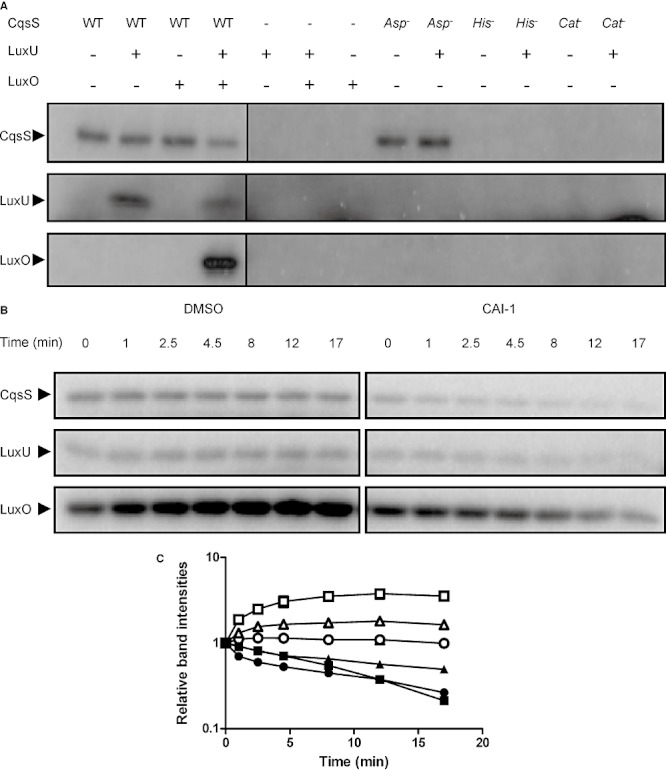
*In vitro* reconstitution of the CqsS → LuxU → LuxO phosphorylation cascade. A. Auto-phosphorylation of CqsS and phosphotransfer to LuxU and to LuxO were assayed with wild-type CqsS, CqsS *His^-^*, CqsS *Cat^-^* and CqsS *Asp^-^*. All phosphorylated proteins were run on the same gel and extracted. Only the relevant regions are shown for simplicity. B. A phosphorylation reaction was carried out with CqsS, LuxU and LuxO for 1 min and divided in half. One half was supplemented with DMSO (left) and the other with 500 µM CAI-1 (right). Samples were taken at the indicated time points. The top, middle and bottom rows show the phosphorylated CqsS, LuxU and LuxO proteins respectively. C. Experiments in (B) were performed in duplicate. Band intensities for CqsS∼P (circles), LuxU∼P (triangles) and LuxO∼P (squares) when DMSO was added (open symbols) and when CAI-1 was added (closed symbols) were normalized to the time zero level of each protein.

In order to investigate how CAI-1 binding regulates QS signalling events, we undertook a biochemical approach to reconstitute the CqsS → LuxU → LuxO circuit *in vitro*. We cloned and overexpressed the CqsS receptor in recombinant *Escherichia coli*, and examined *in vitro* auto-phosphorylation of CqsS (H1) using inverted membrane vesicles and [γ-^32^P] ATP. To verify the prediction that His194 in the DHp domain is the site of phosphorylation, we constructed a CqsS H194Q mutant (called CqsS *His*^-^). In our assay, wild-type CqsS exhibits auto-phosphorylation; however, CqsS *His^-^* does not (Fig. 2A, top row). Several conserved glycine residues in the CA domain are predicted to be critical for ATP binding. To test this assumption, the mutant CqsS G379A/G381A (called CqsS *Cat^-^*) was constructed and it too is incapable of auto-phosphorylation (Fig. 2A, top row). Finally, and also as expected, when the predicted phosphoryl receiving aspartate residue Asp618 in the attached receiver domain (D1) is mutated to Asn, the resulting mutant CqsS D618N (CqsS *Asp^-^*) remains capable of auto-phosphorylation (Fig. 2A, top row).

Genetic analyses imply that, at low cell density, CqsS is a kinase that transfers the phosphoryl group to the HPt protein LuxU, and LuxU, in turn, transfers the phosphoryl group to the response regulator LuxO. To examine this *in vitro*, LuxU and LuxO were overexpressed and purified from *E. coli*. LuxU phosphorylation occurred when both CqsS and LuxU proteins were present and no LuxU phosphorylation occurred in the absence of CqsS (Fig. 2A, middle row). Neither the CqsS *His^-^* nor the CqsS *Cat^-^* protein could transfer phosphoryl groups to LuxU. Likewise, the CqsS *Asp^-^* mutant, although active for auto-phosphorylation, does not transfer the phosphoryl group to LuxU, presumably due to the participation of Asp618 in the phosphorelay shuttle (Freeman *et al*., 2000) (Fig. 2A, middle row). We next turned our attention to LuxO. LuxO phosphorylation was observed only when wild-type CqsS, LuxU and LuxO proteins were present together (Fig. 2A, bottom row and Fig. S1). Thus, CqsS cannot transfer phosphoryl groups to LuxO directly; LuxU is required. Exactly analogous to what we found for LuxU, defects in any of the key CqsS residues (CqsS *His^-^*, CqsS *Cat^-^* and CqsS *Asp^-^*) eliminated phosphotransfer to LuxO (Fig. S1).

These preliminary experiments establish the CqsS → LuxU → LuxO pathway, putting us in position to examine whether we could exploit having the CAI-1 ligand in hand to investigate what happens to a sensor histidine kinase when it encounters its stimulus. Genetic evidence indicates that CAI-1 binding switches CqsS from a kinase to a phosphatase, resulting in the dephosphorylation of phosphorylated LuxU (LuxU∼P) and phosphorylated LuxO (LuxO∼P). To examine if this switch can occur *in vitro*, we incubated CqsS, LuxU, LuxO and radioactive ATP together for 1 min. The reaction was then divided in half and either DMSO or CAI-1 was added. In the experiment in which DMSO was supplied, the CqsS∼P did not change while LuxU∼P and LuxO∼P levels increased initially and then remained constant over time, indicating continuous phospho-flow from CqsS to LuxU and to LuxO until the system reached steady state (Fig. 2B, left, Fig. 2C). However, the levels of all of the phosphorylated proteins decreased dramatically when CAI-1 was supplied (Fig. 2B, right, Fig. 2C), indicating that CAI-1 transforms CqsS from a kinase to a phosphatase. These results are not due to the instability of LuxU∼P and/or LuxO∼P because both LuxU∼P and LuxO∼P are stable (half life > 10 min) in our experimental set-up. Also, the CAI-1 molecule does not destabilize LuxU∼P or LuxO∼P (Fig. S2).

### Receptor-ligand specificities are maintained *in vitro*

**Fig. 3:**
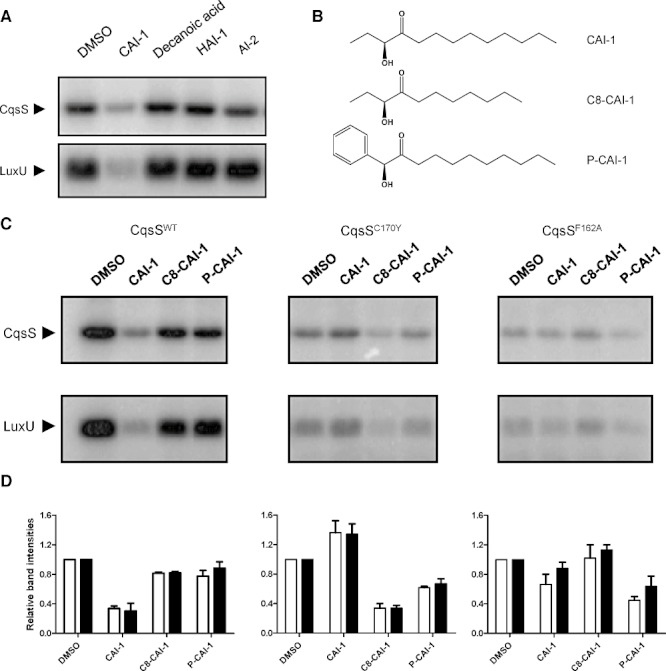
Receptor-ligand specificities are maintained *in vitro*. A. CqsS and LuxU phosphorylation were examined in the presence of DMSO or 100 µM CAI-1, Decanoic acid, HAI-1 (3-hydroxy-C4-HSL), or AI-2 [(2*S*,4*S*)-2-methyl-2,3,4-tetrahydroxytetrahydrofuran-borate]. Both phosphorylated proteins were run on the same gel and extracted. Only the relevant regions are shown for simplicity. B. Structures of CAI-1, C8-CAI-1 and P-CAI-1. C. CqsS auto-phosphorylation and phosphotransfer to LuxU were examined with the wild-type CqsS receptor (left), the CqsS^C170Y^ (middle) and the CqsS^F162A^ (right) mutant receptors in the presence of DMSO or 100 µM of CAI-1, C8-CAI-1 or P-CAI-1. Reactions were carried out for 30 s. Both phosphorylated proteins were run on the same gel and extracted. Only the relevant regions are shown for simplicity. D. Experiments in (C) were performed in duplicate and band intensities were normalized to the respective DMSO control in each set of experiments. White bars and black bars show the quantification for CqsS and LuxU respectively.

We wondered if the above result is specific to the CAI-1 ligand or if any ligand could convert recombinant CqsS from a kinase to a phosphatase. To examine this, we tested decanoic acid, the *Vibrio harveyi* autoinducer HAI-1, and AI-2 for the ability to convert CqsS to a phosphatase. Decanoic acid resembles the fatty acid tail of CAI-1 but lacks the α-hydroxy ketone head group. HAI-1 is the major *V. harveyi* autoinducer and AI-2 is the autoinducer of the *V. cholerae* LuxPQ QS pathway (Chen *et al*., 2002; Henke and Bassler, 2004). HAI-1 and AI-2, while being autoinducers, do not share structural similarity to CAI-1. None of these molecules decreased the levels of CqsS∼P or LuxU∼P (Fig. 3A), indicating that CqsS remains a kinase in the presence of these molecules, and therefore CAI-1 modulation of CqsS kinase activity is specific.

In a previous report, we identified ‘gatekeeper’ residues in the CqsS transmembrane domain that constrain the CAI-1 structure allowable for agonism (i.e. conversion of CqsS from a kinase to a phosphatase) (Ng *et al*., 2010). Specifically, the CqsS^C170Y^ receptor is not agonized by CAI-1. However, C8-CAI-1 (Fig. 3B), which has a carbon tail that is two carbons shorter than CAI-1, is a CqsS^C170Y^ agonist. The CqsS^F162A^ receptor also does not respond to CAI-1. Rather, it responds to Phenyl-CAI-1 (P-CAI-1) (Fig. 3B), a synthetic molecule with an enlarged head group compared to CAI-1. To test if these receptor-ligand specificities are maintained *in vitro*, inverted membranes containing the mutant CqsS^C170Y^ and CqsS^F162A^ receptors were prepared. CqsS phosphorylation and phosphotransfer to LuxU were examined with DMSO, CAI-1, C8-CAI-1 or P-CAI-1. Consistent with the results in Figs 2 and 3A, the wild-type receptor was converted from a kinase to a phosphatase by CAI-1, as indicated by decreased CqsS∼P and LuxU∼P compared with the DMSO control (Fig. 3C and D, left). We note that modest CqsS phosphorylation and phosphotransfer to LuxU does occur in the presence of CAI-1, indicating that there remains residual kinase activity despite ligand binding. This result is consistent with the observation that, *in vivo*, even at high cell density, when CAI-1 concentration is high, low levels of *qrr*1-4 transcripts can be detected (Rutherford *et al*., 2011). In contrast to CAI-1, neither C8-CAI-1 nor P-CAI-1 reduced the wild-type CqsS∼P and LuxU∼P levels compared with the DMSO control (Fig. 3C, left). These results show that, at the concentrations tested, neither C8-CAI-1 nor P-CAI-1 functions as an agonist of the wild-type CqsS receptor. At higher concentrations, C8-CAI-1 does reduce the wild-type CqsS∼P and LuxU∼P levels (see below). Thus, C8-CAI-1 is a weak agonist of CqsS, consistent with earlier genetic studies (Ng *et al*., 2011).

While the wild-type receptor does not respond to the CAI-1 analogues, this is not the case for the receptors containing the gate keeper mutations (Fig. 3C and D, middle and right). Whereas CqsS phosphorylation and LuxU phosphorylation are weaker overall for the mutant CqsS^C170Y^ and CqsS^F162A^ receptors compared with the wild-type receptor, their ligand specificities can nonetheless be examined. In the case of CqsS^C170Y^, CAI-1 did not significantly alter CqsS^C170Y^ phosphorylation and LuxU phosphorylation. However, when C8-CAI-1 was added, CqsS^C170Y^ phosphorylation and LuxU phosphorylation decreased (Fig. 3C and D, middle). Surprisingly, P-CAI-1 inhibited CqsS^C170Y^ phosphorylation and LuxU phosphorylation but to a lesser extent than C8-CAI-1. This weak P-CAI-1 agonist activity towards the CqsS^C170Y^ receptor has not been observed previously; however, lower concentrations were used in the earlier *in vivo* experiments. These results show that C8-CAI-1, but not CAI-1, functions as a strong agonist of the CqsS^C170Y^ receptor and P-CAI-1 functions as a weak agonist. Regarding the CqsS^F162A^ receptor, CqsS^F162A^ phosphorylation and LuxU phosphorylation decreased only in the presence of P-CAI-1 but not when CAI-1 or C8-CAI-1 was added (Fig. 3C and D, right), indicating that CqsS^F162A^ is specific for P-CAI-1. Thus, the specific *in vivo* interactions between ligands and receptor transmembrane sensing domains and their effects on signal transduction are maintained *in vitro*.

### CqsS antagonism occurs *in vitro*

**Fig. 4:**
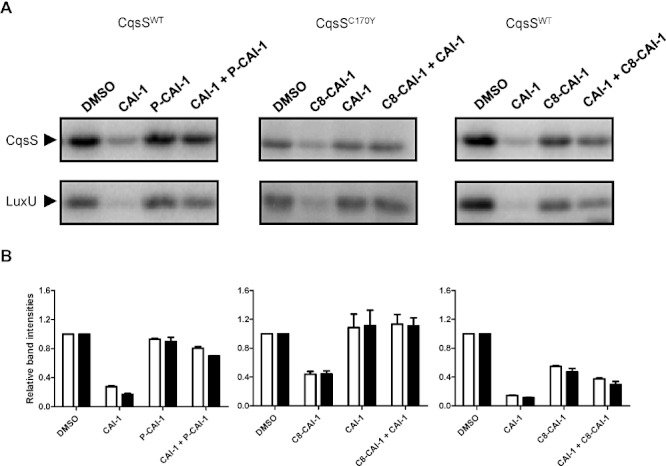
CqsS antagonism occurs *in vitro*. Antagonism was examined with the CqsS wild-type receptor (left and right) and the CqsS^C170Y^ mutant receptor (middle). Reactions containing LuxU and either wild-type CqsS or CqsS^C170Y^ were supplemented with the ligand(s) indicated above each lane. Agonists were present at 10 µM while antagonists were present at 100 µM (left and middle) or 500 µM (right). Reactions were carried out for 30 s. Both phosphorylated proteins were run on the same gel and extracted. Only the relevant regions are shown for simplicity. B. Experiments in (A) were performed in duplicate and band intensities were normalized to the respective DMSO control in each set of experiments. White bars and black bars show the quantification for CqsS and LuxU respectively.

We wondered if we could adapt our *in vitro* system to study antagonism of the CqsS receptor. *In vivo*, P-CAI-1 antagonizes the CAI-1 activity of wild-type CqsS. When both CAI-1 and P-CAI-1 were added to membranes containing wild-type CqsS, phosphorylation of CqsS and LuxU were stronger than when CAI-1 was present alone. This result shows that the CAI-1 agonist activity is antagonized by P-CAI-1 (Fig. 4A and B, left). To confirm that antagonism *in vitro* applies to more than one pair of molecules and a single receptor, we expanded our investigation to the CqsS^C170Y^ receptor. As a reminder, the CqsS^C170Y^ receptor is agonized by C8-CAI-1 and antagonized by CAI-1 (Ng *et al*., 2010). This holds true *in vitro*: phosphorylation of CqsS^C170Y^ and LuxU is significantly stronger in the presence of both CAI-1 and C8-CAI-1 compared with C8-CAI-1 alone (Fig. 4A and B, middle).

We have previously proposed a theoretical two-state model for histidine kinases (Swem *et al*., 2008). In our model, the kinase domain of a receptor like CqsS exists in two states, a ‘kinase on’ state and a ‘kinase off’ state (Ng *et al*., 2010). Agonists, like CAI-1, preferentially bind and stabilize the ‘kinase off’ state. Because CqsS also possesses phosphatase activity, it appears to be a phosphatase in the presence of an agonist such as CAI-1. A weak agonist also prefers to bind and stabilize the ‘kinase off’ state. However, a weak agonist exhibits reduced preference for the ‘kinase off’ state compared with a strong agonist. The two-state model predicts that when a weak agonist and a strong agonist are present together, the weak agonist will functionally behave as an antagonist due to competition for the ligand binding site. In such a scenario, the receptor will have a higher probability to be in the ‘kinase on’ state when both molecules are present, compared with when the stronger agonist is present alone. Having CqsS functioning *in vitro* and a set of agonists with different potencies allows us to explicitly test our model *in vitro*. First, when high concentration of C8-CAI-1 was applied, decreased CqsS and LuxU phosphorylation were observed, consistent with its weak agonist identity (Fig. 4A and B, right). Next, we combined C8-CAI-1, a weak agonist of CqsS, with CAI-1, a strong agonist of CqsS, and examined CqsS and LuxU phosphorylation. When C8-CAI-1 was present together with CAI-1, CAI-1-mediated inhibition of phosphorylation of CqsS and LuxU was reduced (Fig. 4, right). Therefore, C8-CAI-1 is a weak agonist of CqsS when it acts alone but it can also act as an antagonist in the presence of CAI-1, validating the predictions of the theoretical two-state model.

### CAI-1 regulates CqsS His194 auto-phosphorylation

**Fig. 5:**
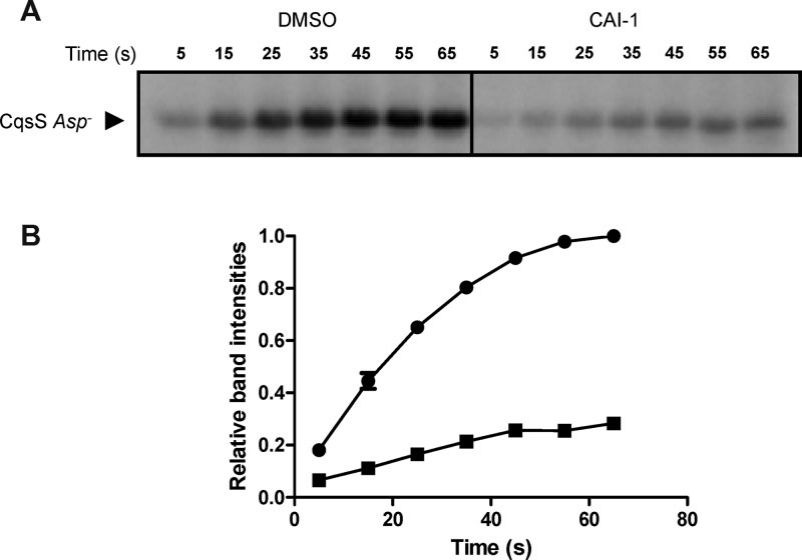
CAI-1 regulates CqsS auto-phosphorylation. A. CqsS auto-phosphorylation was examined using the CqsS *Asp^-^* mutant construct in the presence of DMSO (left) or 500 µM CAI-1 (right). Samples were taken at the indicated time points. B. Experiments in (A) were performed in duplicate. Band intensities from the DMSO control (circles) and CAI-1 treated (squares) samples were quantified and normalized to the 65 s time point band from the DMSO control in each experiment.

CAI-1 driven kinase inhibition of CqsS could be due to regulation of CqsS His194 auto-phosphorylation, phosphotransfer to Asp618, phosphotransfer from Asp618 to LuxU His58, or CAI-1 could control the CqsS phosphatase activity. To pinpoint which step(s) is controlled by CAI-1, we first tested if His194 auto-phosphorylation is regulated by CAI-1 using the CqsS *Asp^-^* receptor construct. Our rationale is that the CqsS *Asp*^-^ construct can auto-phosphorylate but all subsequent phosphotransfer processes are eliminated. Thus, this construct allows us to exclusively examine the dependence of His194 auto-phosphorylation on CAI-1. Rapid auto-phosphorylation of the CqsS *Asp^-^* mutant construct occurred when it was incubated with [γ-^32^P] ATP, and maximum auto-phosphorylation was achieved at 1 min (Fig. 5A and B). Addition of CAI-1 decreased the initial phosphorylation rate sixfold (Fig. 5A and B). These results show that His194 auto-phosphorylation is indeed regulated by CAI-1. This result does not preclude CAI-1 regulation of other phosphotransfer events, and we address this in the subsequent sections.

### Phosphotransfer processes are not affected by CAI-1

**Fig. 6:**
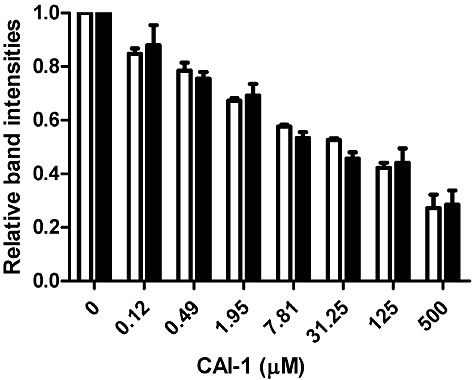
Phosphotransfer steps subsequent to CqsS auto-phosphorylation are not CAI-1 regulated. Auto-phosphorylation of His194 on CqsS *Asp^-^* and phosphotransfer from wild-type CqsS to LuxU were measured in the presence of various concentrations of CAI-1. Reactions were carried out for 30 s. Experiments were performed in duplicate and band intensities of phosphorylated CqsS *Asp^-^* (white bars) and LuxU (black bars) were quantified and normalized to their respective DMSO controls.

In addition to this first phosphorylation step, downstream phosphotransfer processes (His194∼P to Asp618 within CqsS and/or CqsS Asp618∼P to LuxU His58) could also be inhibited by CAI-1 binding. We reason that if His194 auto-phosphorylation is the only step that is controlled by CAI-1, then CAI-1 inhibition of LuxU His58 phosphorylation should track with inhibition of CqsS His194 auto-phosphorylation. By contrast, if either or both of the subsequent phosphotransfer steps, His194∼P to Asp618 in CqsS or CqsS Asp618∼P to LuxU His58, is CAI-1 regulated, following CAI-1 addition, inhibition of LuxU His58 phosphorylation should be more severe than inhibition of CqsS His194 auto-phosphorylation. To assess this, we compared the extent of CqsS His194 auto-phosphorylation in the CqsS *Asp^-^* construct with that of LuxU phosphorylation by the wild-type CqsS receptor. When different concentrations of CAI-1 were added, both His194∼P on the CqsS *Asp^-^* construct and phosphotransfer to His58∼P on LuxU from wild-type CqsS were inhibited to the same extent (Fig. 6). These results suggest that CAI-1 does not affect phosphotransfer processes other than the initial His194 auto-phosphorylation.

### CqsS phosphatase activity is independent of CAI-1

**Fig. 7:**
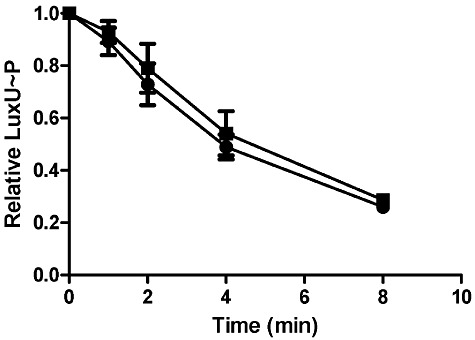
The CqsS phosphatase activity is not regulated by CAI-1. Phosphorylated LuxU was purified and divided in half. DMSO (circles) or 500 µM CAI-1 (squares) was added to one half of the sample. CqsS was added to both reaction mixtures at time zero and aliquots were subsequently removed at the indicated time points. Experiments were performed in duplicate and band intensities were normalized to the time zero band from each experiment.

In addition to kinase activity, CqsS possesses a phosphatase activity that dephosphorylates LuxU∼P. We tested if this activity is regulated by CAI-1. To obtain LuxU∼P, LuxU was first phosphorylated by CqsS. Subsequently, CqsS was removed by ultracentrifugation and ATP was removed by gel filtration. To determine which region of CqsS contains the phosphatase activity, we assayed LuxU∼P dephosphorylation by CqsS *His^-^*, CqsS *Cat^-^* and CqsS *Asp^-^*. Both CqsS *His^-^* and CqsS *Cat^-^* could dephosphorylate LuxU∼P, indicating that His194 or the CqsS catalytic site is not required for CqsS phosphatase activity (Fig. S3). By contrast, CqsS *Asp^-^* was incapable of dephosphorylation of LuxU∼P, suggesting that Asp618 in the CqsS receiver domain is essential for the CqsS phosphatase activity (Fig. S3). To test for CAI-1 regulation of phosphatase activity, the wild-type CqsS was used to dephosphorylate LuxU∼P in the presence and absence of CAI-1. There was no difference between dephosphorylation of LuxU∼P with and without CAI-1 (Fig. 7), demonstrating that CAI-1 does not regulate the CqsS phosphatase activity.

### CAI-1 binding causes a conformational change

**Fig. 8:**
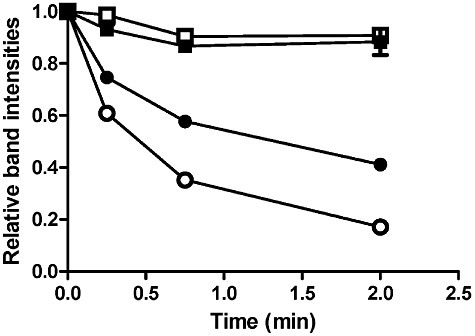
CAI-1 affects dephosphorylation of CqsS His∼P by ADP. 100 µM ADP was added to phosphorylated CqsS *Asp^-^* together with DMSO (open circles) or 500 µM CAI-1 (closed circles). At that time of ADP and ligand addition, 1 mM ATP was also added to terminate new labelling. Aliquots were removed at the indicated time points. The same experiment was performed with 100 µM EDTA present together with DMSO (open squares) or CAI-1 (closed squares). Experiments were performed in duplicate and band intensities were normalized to the time zero band from each experiment.

To explore the mechanism by which CAI-1 binding inhibits His194 auto-phosphorylation, we relied on a phenomenon discovered in studies of the chemotaxis TCS system showing that the phosphorylated histidine residue of the CheA sensor kinase could be dephosphorylated by ADP (Tawa and Stewart, 1994). Thus, in CheA, the His∼P is accessible to ADP. We investigated whether CqsS His194∼P is also capable of being dephosphorylated by ADP and whether CAI-1 binding alters this reaction. To do this, we used the CqsS *Asp^-^* construct because it is functional for the initial His194 auto-phosphorylation step but further phosphotransfer processes do not occur (Fig. 2A). Following auto-phosphorylation, the ^32^P labelled His194∼P rapidly decreased when ADP was added, showing that CqsS His194 is dephosphorylated by ADP (Fig. 8). However, ^32^P labelled His194∼P decreased more slowly when CAI-1 was added together with ADP, indicating that dephosphorylation of His∼P is inhibited by CAI-1 (Fig. 8). In the presence of EDTA, the His∼P remained stable when either CAI-1 or DMSO was included (Fig. 8), indicating the importance of Mg^2+^ and presumably the ATP binding domain, for the dephosphorylation process to occur. We conclude that CAI-1 binding to the CqsS transmembrane domain results in a conformational change that protects the His194∼P from being accessed by the catalytic ATP binding domain.

## Discussion

Bacterial TCSs play crucial roles in environmental adaptation. Although many model TCSs are well established, how their activities are controlled by stimuli has not, in general, been rigorously investigated, primarily due to the profound lack of identified ligands and the difficulties encountered in biochemical and structural studies of membrane spanning proteins (Lee *et al*., 1999; Timmen *et al*., 2006; Cheung and Hendrickson, 2010). In addition, TCSs containing many components are difficult to reconstitute *in vitro*. Even the paradigmatic bacterial chemotaxis TCS has not been examined in a completely reconstituted system *in vitro*. This is due to the fact that the membrane bound chemotaxis receptors (e.g. Tar) are not histidine kinases; rather, these receptors regulate the activity of the soluble histidine kinase CheA (Hazelbauer and Lai, 2010). Second, the chemotaxis system relies on the specific organization of the receptors in arrays (Webre *et al*., 2004). Third, chemotaxis signalling is subject to multiple types of regulation such as methylation of the receptor by CheR, demethylation by CheB, and dephosphorylation of the response regulator CheY by the phosphatase CheZ (Ninfa *et al*., 1991). In *V. cholerae*, by contrast, QS relies on a three protein phosphorelay system to detect the major autoinducer CAI-1 (Miller *et al*., 2002). Moreover, the structure of the ligand CAI-1 is known, and CAI-1 and analogues are readily available (Kelly *et al*., 2009; Ng *et al*., 2010; Bolitho *et al*., 2011; Wei *et al*., 2011). Finally, a panel of CqsS ‘gatekeeper’ mutants, with altered ligand specificities, has been engineered (Ng *et al*., 2010). Thus, the CqsS system is tractable to mechanistic dissection *in vitro*. Using inverted *E. coli* membranes containing the full-length CqsS receptor together with purified downstream protein components, we were able to reconstitute the *V. cholerae* QS phosphorylation pathway *in vitro*. The complete phosphorylation cascade was established from CqsS, the hybrid sensor histidine kinase, through LuxU, the HPt protein, to LuxO, the response regulator. Our set of sensing mutants and ligand analogues allowed us to discover that ligand-receptor specificities are maintained *in vitro*, and that CAI-1 binding regulates His194 auto-phosphorylation, whereas phosphotransfer and phosphatase activity are not affected by ligand binding.

It is interesting that QS relies on a phosphorelay system rather than a simple TCS system. We speculate that having the HPt protein LuxU provides a hub for signal integration. Indeed, *V. cholerae* QS relies on two parallel sensory pathways. In addition to the CAI-1/CqsS pathway, input from the AI-2/LuxPQ sensory pathway converges with that from CAI-1/CqsS at LuxU (Miller *et al*., 2002). Likewise, in the related bacterium *V. harveyi*, information from three hybrid sensor histidine kinases, CqsS, LuxPQ and LuxN all converge at *V. harveyi* LuxU (Henke and Bassler, 2004). The particular domain architecture of the *Vibrio* QS pathway ensures that the enzymatic activities required for phosphotransfer to and dephosphorylation of LuxU are colocalized within the same protein (CqsS, LuxQ and LuxN). Therefore, modulation of receptor levels potentially regulates phospho-flow in both directions. Feed-back regulation of QS receptor levels has been demonstrated in *V. harveyi* (Teng *et al*., 2011). We speculate that *V. cholerae* CqsS could also be subject to similar feed-back regulation.

Proteins like CqsS that contain both the H1 and D1 modules are common in bacteria. Some other bacterial hybrid histidine kinases have domain arrangements similar to ArcB and BvgS, which contain the H1, D1 and the H2 domains (Iuchi *et al*., 1990; Uhl and Miller, 1996). ArcB responds to the redox state of the membrane and anaerobic metabolites, while BvgS responds to temperature, SO_4_^2–^, and nicotinic acid. CqsS, interestingly, resembles the domain architectures of sensory proteins present in eukaryotic organisms. In *Arabidopsis thaliana*, over 10 sensor histidine kinases have been identified, all of which contain the histidine kinase domain (H1) and an attached receiver domain (D1) (Mizuno, 2005). While most of these sensor histidine kinases have unknown functions, interestingly, in *A. thaliana*, detection of ethylene, a plant hormone that controls growth and development, relies on the hybrid sensor histidine kinase ETR1 that resembles CqsS (Voet-van-Vormizeele and Groth, 2008; Kim *et al*., 2011). Response to another *A. thaliana* master growth hormone, cytokinin, requires a phosphorelay system consisting of several hybrid sensor histidine kinases CRE1, AHK2 and AHK3 (H1, D1), multiple HPt proteins called APHs (H2), and multiple response regulators called ARRs (D2) (Inoue *et al*., 2001; Sheen, 2002). It is striking to us that ‘hormone-like’ signalling molecules in both bacteria and plants are detected by similarly arranged sensory systems. We wonder if a fundamental advantage of these systems is in signal integration. HPt proteins linking sensor histidine kinases and response regulators might serve as ideal merging points for inputs from multiple receptors.

Our work shows that while histidine auto-phosphorylation is inhibited by CAI-1, the CqsS phosphatase activity is not regulated by the ligand. Having a constant CqsS phosphatase activity could be crucial for properly timed QS transitions. During the low cell density to high cell density transition, although CAI-1 inhibition of the CqsS kinase ensures that little new LuxO∼P is generated, existing LuxO∼P could still activate transcription, due to its high stability (Fig. S2). However, because CqsS possesses a constant phosphatase activity, it ensures rapid dephosphorylation of any existing LuxO∼P. During the high cell density to low cell density transition, the constant CqsS phosphatase activity would not significantly affect LuxO∼P generation due to the much stronger CqsS kinase activity. Thus, rapid phosphorylation of LuxU and LuxO occurs when CAI-1 disappears.

His194 auto-phosphorylation is inhibited by CAI-1. This result is consistent with previously proposed models suggesting that TCS binding of ligands triggers downstream conformational changes in sensor histidine kinases that affect interactions between the catalytic domains and the histidines in the DHp domains (Borkovich and Simon, 1990; Neiditch *et al*., 2006). We verified this idea by showing that dephosphorylation of His194∼P by ADP is slower in the presence of CAI-1, suggesting that binding of CAI-1 in the transmembrane domain causes a conformational change that repositions the His194 on the DHp domain away from the catalytic domain. Therefore, in the presence of CAI-1, new rounds of phosphorylation are inhibited. Additionally, already phosphorylated His194 also becomes less accessible to the catalytic domain, resulting in its higher stability compared with in the absence of the ligand.

Our previous structural study of an analogous QS receptor, LuxPQ, suggests that upon ligand binding, a symmetry-breaking conformational change occurs in the periplasm that is transduced across the membrane to alter the relative positions of the histidine in the DHp domain and the catalytic domain active site (Neiditch *et al*., 2006). However, in the LuxPQ case, two proteins are required for transducing the AI-2 autoinducer signal. LuxP, the periplasmic autoinducer binding protein, binds AI-2 and induces the conformational change in the partner hybrid sensor kinase LuxQ through protein–protein interactions (Neiditch *et al*., 2005; 2006). Extensive studies with CheA have led to a similar model (Borkovich and Simon, 1990). In the CheA case, the chemotaxis receptor Tar is membrane associated and it regulates the cytoplasmic kinase CheA. When the ligand binds to the Tar receptor, it is proposed to induce a ‘closed’ confirmation of the CheA kinase. It is curious to us that three distinct TCSs with three different modular domain architectures use a related mechanism for regulating kinase activity. Evolution has produced a variety of domain arrangements for histidine kinases in TCSs, yet; at least in those cases examined, the underlying regulation mechanism seems to be conserved.

In our current study, P-CAI-1, a CqsS antagonist, also antagonizes CqsS in the presence of CAI-1 *in vitro*. In addition, C8-CAI-1, a weak agonist of CqsS, also acts as an antagonist of CqsS in the presence of CAI-1 *in vitro*. This result is consistent with our two-state model (see Results). Competition between a weak agonist and a strong agonist for the ligand binding pocket results in an intermediate effect rather than a synergistic effect. Antagonism can also be replicated *in vitro* with the CqsS^C170Y^ mutant receptor, which possesses altered specificity for ligands. *In vitro*, C8-CAI-1 is a strong agonist and CAI-1, which does not affect CqsS^C170Y^ when acting alone, is an antagonist of the CqsS^C170Y^ receptor. Therefore, CAI-1 likely binds and stabilizes the ‘kinase on’ and ‘kinase off’ states of CqsS^C170Y^ receptor with equal preference, and thus appears neutral in the absence of other molecules. Alternatively, to function as an antagonist, CAI-1 could prefer to bind and stabilize the ‘kinase on’ state of CqsS^C170Y^. In either case, CAI-1 certainly competes for the binding site with C8-CAI-1 and therefore acts functionally as an antagonist. Thus, predictions of the two-state theoretical model are consistent with the results of the experiments using our *in vitro* phosphorylation system.

Bacteria exist in niches containing complex microbial-species compositions. Thus, the chemical environments they encounter contain numerous signalling cues, including many classes of autoinducers, the concentrations of which change in time and in space. In a simplified system, we have examined receptor histidine kinase regulation by a set of agonists and antagonists. To the best of our knowledge, this study shows, for the first time, that histidine auto-phosphorylation of the sensor is the only step that is regulated by the ligands. Structural studies of apo-CqsS and the CqsS receptor bound to agonists and antagonists should reveal the conformational changes induced by these ligands. Additionally, future work with reconstituted circuits that contain both the CqsS and LuxPQ sensors will be useful to understand QS signal integration.

## Experimental procedures

### Cloning of CqsS, LuxU and LuxO

DNA manipulations were performed using standard methods (Sambrook *et al*., 1989). The gene encoding CqsS was PCR amplified from *V. cholerae* genomic DNA and cloned into the pET21b vector. Plasmids encoding CqsS H194Q (CqsS *His^-^*), CqsS D618N (CqsS *Asp^-^*), CqsS G379A/G381A (CqsS *Cat^-^*), CqsS^C170Y^ and CqsS^F162A^ were constructed from the plasmid containing wild-type CqsS using the Quikchange II XL Site-Directed Mutagenesis Kit (Stratagene). The genes encoding LuxU and LuxO were PCR amplified from *V. cholerae* genomic DNA and cloned into the pET28b vector. We call the LuxO site of phosphorylation D47 to be consistent with earlier publications and to match reported nomenclature of mutants. As part of the present work, we discovered the *in vivo* LuxO translational start site and found it is 14 codons 5′ to the previously reported start site. Thus, the phosphorylated Asp residue is amino acid 61 in the LuxO protein.

### Preparation of inverted membranes

*Escherichia coli* BL21 (DE3) harbouring plasmids encoding wild type or mutant CqsS constructs were grown with shaking in Luria–Bertani (LB) with 100 µg ml^−1^ kanamycin at 37°C. Overnight bacterial cultures were diluted 1:100 into fresh LB medium with kanamycin. After growth with aeration for 3 h at 37°C, protein production was induced with 1 mM IPTG. The cultures were shifted to room temperature and grown for an additional 5 h with shaking. Cells were harvested by centrifugation, resuspended in lysis buffer (50 mM Tris pH 8.0, 200 mM NaCl, 5 mM β-mercaptoethanol, 5 mM MgCl_2_ and 20 mM imidazole), and lysed under 15 000 psi. Cell lysates were centrifuged at 9300 *g* for 30 min and the cleared fluids were harvested and subjected to ultra-centrifugation at 180 000 *g* for 1 h. Membrane pellets were resuspended in kinase buffer [50 mM Tris pH 8.0, 100 mM KCl, 5 mM MgCl_2_, and 10% (v/v) glycerol] and quantified by SDS-PAGE and Western blot using CqsS purified in detergent as the standard.

### Purification of LuxU and LuxO

*Escherichia coli* BL21 (DE3) containing plasmids encoding LuxU or LuxO were grown and lysed using the above conditions. Cell lysates were centrifuged at 21 000 *g* for 30 min and the cleared fluids were harvested and passed through 5 ml Hi-Trap Chelating columns (GE healthcare) pre-charged with Ni^2+^ ion. Following a wash with 30 ml lysis buffer, proteins were eluted with lysis buffer except that imidazole was included at 500 mM. Proteins were examined by SDS-PAGE followed by Coomassie Brilliant Blue staining and found to be ∼ 95% pure. Purified proteins were dialysed against lysis buffer lacking imidazole and concentrated. Protein concentrations were determined using the Bio-rad protein assay.

### Phosphorylation assays

Phosphorylation assays were performed with inverted membranes containing 2 µM wild-type CqsS or CqsS mutant proteins. In assays containing LuxU and/or LuxO, LuxU and LuxO were supplied at 10 µM. Reactions were carried out in phosphorylation buffer [50 mM Tris pH 8.0, 100 mM KCl, 5 mM MgCl_2_, and 10% (v/v) glycerol], and were initiated with the addition of 100 µM ATP and 2 µCi [γ-^32^P]-ATP (from a stock of 3000 Ci mmol^−1^: Perkin Elmer). For experiments with CAI-1 and analogues, compounds were added 10 min before the initiation of the reactions. For the reconstitution of the complete CAI-1/CqsS, LuxU, LuxO circuit, CAI-1 was supplied at 500 µM. Regulation of histidine phosphorylation by CAI-1 was assayed with 500 µM CAI-1. Agonism was tested at 100 µM. Antagonism was tested with 10 µM agonist and 100 µM (for P-CAI-1 antagonism of CqsS^WT^ and CAI-1 antagonism of CqsS^C170Y^) or 500 µM (for C8-CAI-1 antagonism of CqsS^WT^) antagonist as specified. Reactions were incubated at room temperature and terminated with SDS-PAGE loading buffer. Reaction products were separated using SDS-PAGE. Gels were dried at 80°C on filter paper under vacuum, exposed to a phosphoscreen overnight, and subsequently analysed using a Typhoon 9400 scanner and ImageQuant software.

### Dephosphorylation of LuxU∼P

LuxU was phosphorylated for 5 min in reactions with membrane vesicles containing 4 µM CqsS, 125 µM LuxU, 100 µM ATP and 10 µCi [γ-^32^P]-ATP. Subsequently, the membrane vesicles were removed by ultracentrifugation at 180 000 *g* for 40 min. The supernatants containing LuxU∼P were applied to gel filtration spin columns (Probe Quant G-50, GE healthcare) to remove ATP. Dephosphorylation reactions were initiated by adding inverted membranes containing 2 µM CqsS receptor and either 500 µM CAI-1 or DMSO. Aliquots were taken at the indicated time points and analysed as described above.

### Dephosphorylation of His194∼P by ADP

Phosphorylation of the CqsS *Asp^-^* mutant construct was carried out under the conditions described above for 2 min. His∼P dephosphorylation reactions were initiated by the addition of ADP at 100 µM final concentration and either 500 µM CAI-1 or DMSO. ATP was also added at 1 mM together with DMSO or CAI-1 to terminate new labelling of CqsS *Asp^-^*. In experiments with EDTA, 100 µM EDTA was added together with ligand and nucleotides. Aliquots were taken at the indicated time points and analysed as described above.

### Chemical synthesis

Chemical syntheses of CAI-1, C8-CAI-1, P-CAI-1, HAI-1 and AI-2 have been described (Semmelhack *et al*., 2005; Higgins *et al*., 2007; Swem *et al*., 2008; Ng *et al*., 2010). Decanoic acid was purchased from Sigma-Aldrich.
