# Supplementary material for: Ligand and antagonist driven regulation of the Vibrio cholerae quorum-sensing receptor CqsS
Source: Mol Microbiol. 2012 Feb 14;83(6):1095–108. doi: 10.1111/j.1365-2958.2012.07992.x (PMC3310172; doi:10.1111/j.1365-2958.2012.07992.x)
Supplement: Supplementary file 1 [file mmi0083-1095-SD1.pdf]

Supporting information

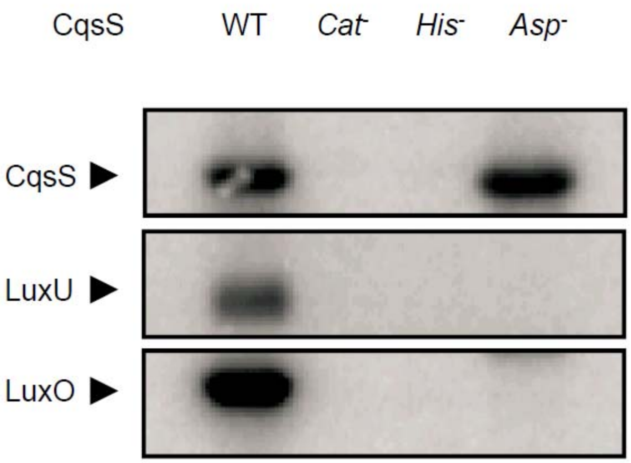

Figure S1. Phosphorylation of LuxO requires wild type CqsS. Phosphotransfer to LuxO was examined from wild type CqsS, CqsS *His*$^{-}$, CqsS *Cat*$^{-}$, and CqsS *Asp*$^{-}$ in the presence of LuxU.

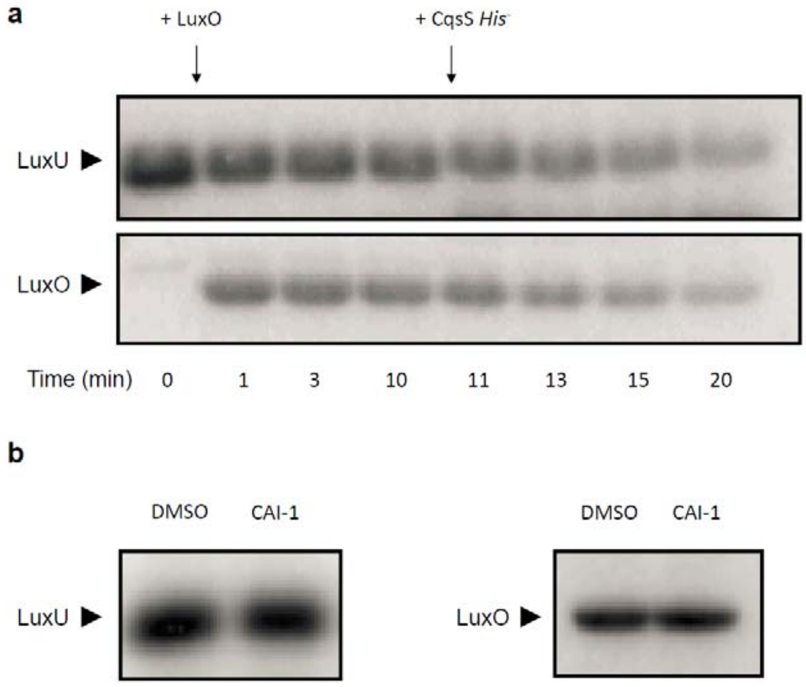

Figure S2. LuxU~P and LuxO~P are stable. (a) Phosphorylated LuxU was purified and is shown in the top left most lane. LuxO was added at T = 0. The bottom row shows phosphotransfer to LuxO. Both LuxU~P and LuxO~P are stable for at least 10 min. Addition of CqsS *His*$^-$ (which is a phosphatase) at T = 10 min shows that the phosphate group can indeed be removed from both LuxU~P and LuxO~P by an active phosphatase mechanism. (b) Left, phosphorylated LuxU was purified and divided in half. Either DMSO or 500 µM CAI-1 was added to one of the aliquots and both samples were analyzed after 10 min. Right, LuxO was phosphorylated for 1 min using purified phosphorylated LuxU. The reaction was divided in half. Either DMSO or 500 µM CAI-1 was added to one of the aliquots and both samples were analyzed after 10 min.

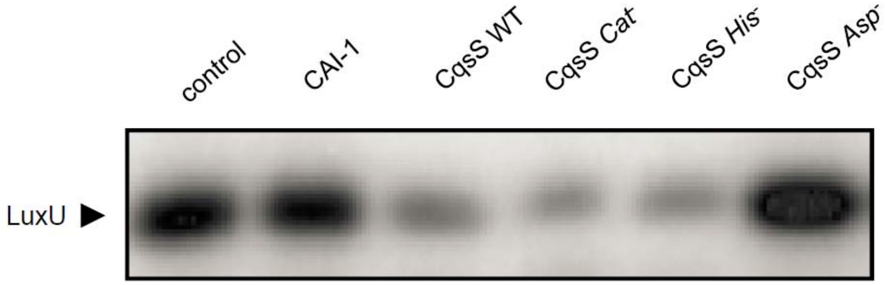

Figure S3. CqsS phosphatase activity does not require His194 or the CA domain, whereas Asp619 is required. Phosphorylated LuxU was purified and is shown in the control lane. Dephosphorylation of LuxU~P was examined following the addition of DMSO, CAI-1, CqsS WT, CqsS *Cat*$^-$, CqsS *His*$^-$, and CqsS *Asp*$^-$ for 10 min.
